# Knowledge and Attitude towards Clinical Trials among General Population of Northern Saudi Arabia during COVID-19 Era: A Cross-Sectional Study

**DOI:** 10.3390/healthcare11050680

**Published:** 2023-02-25

**Authors:** Mohamed Abouelkheir, Ahmed E. Taha, Ashokkumar Thirunavukkarasu, Wesam Saad S. Alkhamsan, Fahd Khalid S. Almutairi, Ali Awadh A. Alanazi, Abdulaziz Lafi M. Alruwaili, Nasser Saleh Alriwely

**Affiliations:** 1 Department of Pharmacology and Therapeutics, College of Medicine, Jouf University, Sakaka 72388, Aljouf, Saudi Arabia; 2 Microbiology and Immunology Unit, Department of Pathology, College of Medicine, Jouf University, Sakaka 72388, Aljouf, Saudi Arabia; 3 Department of Community and Family Medicine, College of Medicine, Jouf University, Sakaka 72388, Aljouf, Saudi Arabia; 4 College of Medicine, Jouf University, Sakaka 72388, Aljouf, Saudi Arabia; 5 Medical Service Department in Ministry of Health, Arar 91411, Northern Border Province, Saudi Arabia

**Keywords:** clinical trials, participation, knowledge, attitude, new drug, Saudi Arabia

## Abstract

Recruiting and retaining sufficient participants is one of the biggest challenges researchers face while conducting clinical trials (CTs). This is due to the fact of misconceptions and insufficient knowledge concerning CTs among the public. The present cross-sectional study was conducted from April 2021 to May 2022. We evaluated knowledge and attitude among 480 participants using a pretested Arabic questionnaire. The correlation between knowledge and attitude score was tested through Spearman’s correlation test, and the logistic regression test evaluated the associated factors for knowledge and attitude. Of the studied participants, 63.5% were male and belonged to the age group less than 30 years (39.6%). Nearly two-thirds (64.6%) of them had never heard of CT. More than half of the participants had poor knowledge (57.1%) and attitude (73.5%) towards CTs. Participants’ knowledge scores were significantly associated with education level (*p* = 0.031) and previous participation in health-related research (*p* = 0.007). Attitude scores were significantly related to marital status (*p* = 0.035) and the presence of chronic diseases (*p* = 0.008). Furthermore, we found a significant positive correlation between knowledge and attitude scores (*p* < 0.001, Spearman’s rho = 0.329). The present study revealed that most of the study population had poor knowledge and moderate attitudes towards CT. Targeted health education programs at different public places are recommended to improve the public’s knowledge of the importance of CT participation. In addition, exploratory and mixed-methods surveys in other regions of KSA is required to recognize the region-specific health education needs.

## 1. Introduction

Clinical trials (CTs) are a type of research study design that allows participants in one or more health-related interventions to assess the effects on health-related outcomes [1]. CTs are considered the gold standard for identifying therapeutic strategies and diagnostic tests and contribute to one of the highest levels of evidence-based practice among healthcare practitioners [2,3]. Several studies worldwide reported that CTs are a powerful measure that improves healthcare services through evidence-based clinical practice for formulating public health decisions that lead to healthcare improvements [4,5]. Furthermore, the need for CTs during emergencies and pandemics such as COVID-19 was insisted on by numerous researchers [6,7].

Currently, 292,537 trials are registered with a clinical trial registry of the US National Library of Medicine from 209 countries, with a low contribution from the middle east and north Africa (MENA) countries, including the Kingdom of Saudi Arabia (KSA) [8,9,10,11]. The number of clinical trials conducted in the KSA in the past 15 years is scarce, considering a population that exceeds 30 million and a huge annual healthcare budget [9]. To manage the necessary health needs of this region, there is an increase in the need for region-specific clinical trials. Despite the increasing prevalence of lifestyle-related and genetic diseases in these countries, the high growth of the population with an increased demand for medication, MENA countries sponsored less than 1% of global clinical trials. Several factors urge these countries to consider, implement, and conduct their own clinical trials [9,12]. The insufficient number of participants in a clinical trial may have several implications, such as nonsignificant results of important findings and loss of resources spent on planning and conducting the trials, as well as loss of reputation of the institution and investigators due to the fact of failed research [11,13].

An adequate number of participants is essential for conducting an effective CT [8]. Recruiting and the retention of sufficient participants is one of the biggest challenges researchers face while conducting CTs. This scenario also continued during the COVID-19 pandemic [8,13,14,15]. Several recommendations have been made to optimize subject participation in CTs. They are broadly divided into participants (i.e., public and patients) and investigator factors. Of these two categories, participants’ factors, such as their knowledge, attitude, and perceptions towards clinical research, including CTs, significantly impacted low recruitment and participation in CTs [8,13].

A study by Al Lawati et al. in 2018 in Oman found low-level participants’ knowledge of CTs; despite their good attitude, their participation in CTs was very low. The study also stated that less than one-third (31.3%) of participants were aware of CTs [16]. Another study conducted in the KSA by Nedal et al. in 2019 found low mean and attitude scores of the study participants towards CTs. They also reported that most of the participants agreed that CTs could be performed with patients, and only 30.5% of the participants agreed that new drugs and other interventions through CTs could be introduced among healthy people [17]. A recent survey assessing general population knowledge and attitude towards CTs stated that only 7.0% of the participants were aware of ClinicalTrails.gov, and 43.1% knew nothing about CTs [18]. The assessment of the general population’s knowledge and attitude towards CTs will help us to remove the misconceptions about CTs by increasing their knowledge according to the knowledge gap on CTs. Hence, the Saudi general population’s willingness to participate in CTs run by the concerned health authorities could significantly increase. Few studies in the KSA have attempted to assess healthcare workers’ and patients’ knowledge and attitude [19,20]. However, to the best of our knowledge, there are no studies that have assessed the knowledge and attitudes towards CTs among the general public during the COVID-19 era, especially in the northern region of the KSA. Hence, the present study was conducted to assess the knowledge of and attitude towards clinical trials among general population of northern KSA, to identify the factors associated with poor knowledge and attitude among them. Furthermore, we measured the correlation between knowledge and attitude among the study population.

## 2. Materials and Methods

### 2.1. Study Design and Setting

The current analytical cross-sectional study was conducted among the general population of Aljouf Province of the KSA from April 2021 to May 2022. Aljouf Province is situated in the northern part of the KSA with a population of approximately half a million.

### 2.2. Sample Size Estimation and Sampling Method

We used the World Health Organization’s (WHO) sample size calculator with the expected population proportion of 50%, design effect of 1, 95% confidence interval (CI), 5% margin of error, and an 80% study power. Considering these measurements, we concluded that 384 was the minimum participants required for this population-based survey. However, the research team took an additional 25%; thus, the final estimated sample size was 480. Using consecutive sampling methods, we recruited 480 participants for the present study. Using this method, the research team collected data from different public places, such as malls, parks, and supermarkets.

### 2.3. Inclusion and Exclusion Criteria

The present study included participants aged 18 years and over who belonged to Aljouf Province. We excluded those who were less than 18 years of age, healthcare workers, health science college students, and those who were mentally ill.

### 2.4. Data Collection

After ethical approval from the local committee of bioethics from Jouf University (approval no: 06-06-41) and other necessary approvals, the survey team initiated the data collection process. The data collector for the current research visited public places and invited participants as per the inclusion criteria. In order to recruit participants for all days of the week, the number of invited participants per day was limited to 50. We briefed them on the purpose of the study, the participants, their roles in the survey, the benefits, risks, and whom to communicate with for further details. After obtaining their willingness to participate through informed consent, the coinvestigators requested the selected person to fill out the data collection form (Google workspace form) on the research team’s electronic gadgets (mobiles, Tab, etc.). The data collection proforma did not include any identifying details of the participants (deidentified data). Hence, we maintained the anonymity of the participants. Furthermore, we maintained the COVID-19 prevention procedures as given by the concerned authorities. After completing the survey, the research team provided the participants with health education related to CTs. We developed the Arabic version of the data collection form based on open-source pieces of existing literature [16,17,19].

The experts from clinical research, clinical pharmacology, and public health entities extracted and finalized the contents in the first stage (i.e., face and content validity). Next, two bilingual (English–Arabic) experts translated the CT questionnaire into Arabic. Furthermore, another set of nonmedical bilingual persons conducted a back translation process. Finally, the original English version and the back-translated version were compared for similarity. We gave the final Arabic version questionnaire to a randomly selected 30 participants from the general population for pilot testing. The participants from the pilot study population provided feedback to ensure all questions on the data collection form were clear. Furthermore, the pilot study analysis did not find any missing data. The construct validity was assessed with the pilot study’s responses. The Cronbach’s alpha (α) value of the developed questionnaire was 0.81 for knowledge (test–retest, r = 0.89; split-half = 0.91) and 0.86 for the attitude section (test–retest, r = 0.86; split-half = 0.90). Therefore, the team conducted the data collection with the pretested form. The data collection proforma consisted of three parts. The first part inquired about the sociodemographic details; the second and third part parts assessed the participants’ knowledge and attitude towards CTs. The research team followed all COVID-19 infection prevention strategies suggested by the Ministry of Health during the data collection process.

### 2.5. Calculation and Categorization of Knowledge and Attitude Score

The knowledge section consisted of 12 questions in which a participant’s correct score was calculated as 1, and wrong/not sure were scored as 0 (the answer key to the correct/wrong scores were available for the principal investigators and coinvestigators, and it was entered accordingly for statistical analysis). The attitude section consisted of 9 questions in which a participant’s positive answer was calculated as 1, and a no/not sure/negative answer was scored as 0. The total scores were combined and converted to 100 percent. The knowledge and attitude percentages were subclassified into three categories: excellent (≥80% of possible score), moderate (60–79% of possible score), and low (<60% of possible scores). Furthermore, we combined low and medium categories as a single category. We compared them with the excellent category according to Bloom’s criteria, commonly used criteria to categorize the knowledge, attitude, and practice of the public for the health-related surveys [17,21].

### 2.6. Data Analysis

The Statistical Package for Social Sciences (SPSS), version 20.0, was used to enter and analyze the data. Descriptive statistics are presented as the frequency and percentage, and continuous data are depicted as the mean and standard deviation (SD). Knowledge and attitude scores were tested for normal distribution by Shapiro–Wilk analysis. The correlation between knowledge and attitude scores was tested through Spearman’s correlation test. The univariate analysis of the present study was performed through the Chi-square test, and multivariable analysis was executed by the binomial logistic regression analysis. A *p*-value of less than 0.05 was considered to be statistically significant. All statistical tests used in this research were two-tailed.

## 3. Results

We contacted 550 eligible individuals during the data collection period. Of the 550 individuals, 480 eligible participants provided consent to participate in the present survey (response rate = 87.27%). Of the 480 studied individuals, the majority (63.5%) were male, currently married (75.2%), working in public sectors (40.6%), with an education level of university or above (62.7%), and 84.8% of them never participated in any health research (Table 1).

The participants’ knowledge-related responses are depicted in Table 2. Nearly two-thirds (64.6%) of them had never heard of CT, and 56.3% were aware of the Saudi FDA. Regarding the CT implementation process, most participants believed that the research team could initiate CT on patients without written consent, and 65% of the participants responded that the participants could withdraw at any time during the CT process. The mean ± SD score of the knowledge section was 7.10 ± 2.03.

Regarding attitude-related responses, only 24.2% of the respondents agreed to test a new drug on the patients. However, approximately two-thirds (66.7%) of them agreed to test an approved drug on the patients. The majority (88.1%) of the participants denied agreeing to test a new drug on pediatric patients. The mean ± SD of the attitude score was 4.38 ± 2.04 (Table 3).

**Figure 1:**
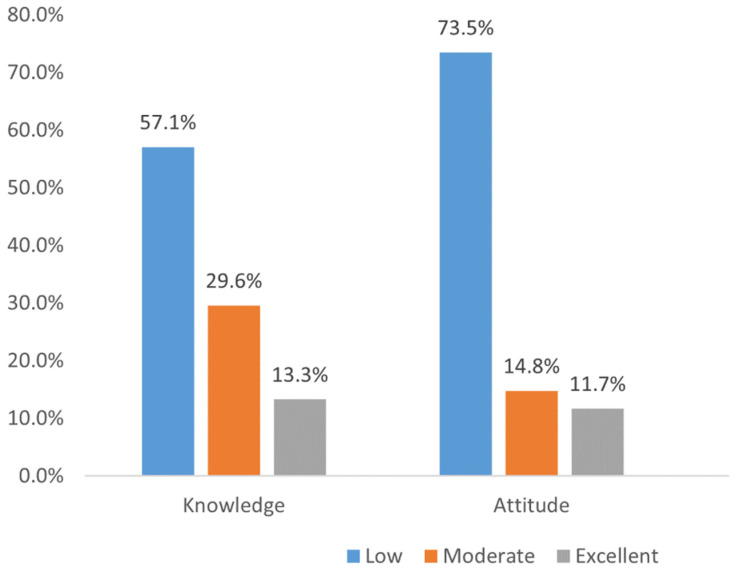
Knowledge and attitude categories (*n* = 480).

Of the studied population, 13.3% and 11.7% had excellent knowledge and attitudes towards CT. The remaining participants belonged to either the poor or moderate categories (Figure 1).

The participants’ background characteristics and their association with the knowledge and attitude scores are presented in Table 4. The participants’ knowledge scores were significantly associated with education level (*p* = 0.006) and their previous participation in health-related research (*p* = 0.005). The attitude scores were significantly related to marital status (*p* = 0.019) and the presence of chronic diseases (*p* = 0.006).

We ran a binary logistic regression to find the categories of factors associated with knowledge and attitude of CTs. After adjusting with the other covariables of the present study, we found that the knowledge category was significantly associated with education level (adjusted odds ratio (AOR) = 1.73, 95% confidence interval (CI) = 1.15–2.84, *p* = 0.031) and previous participation in health research (AOR = 0.63, 95 CI = 0.47–0.89, *p* = 0.007). The attitude category was significantly associated with marital status (AOR = 1.84, 95% CI = 1.17–2.98, *p* = 0.035) and the presence of any chronic diseases (AOR = 0.71, 95% CI = 0.54–0.91, *p* = 0.008) (Table 5).

We found a significant positive correlation (applied test: Spearman’s correlation test) between knowledge and attitude scores (*p* < 0.001, rho = 0.329) (Table 6).

## 4. Discussion

The present study aimed to assess the northern Saudi general population’s knowledge, attitude, and associated factors towards CT. This population-based study revealed that the knowledge regarding CTs was generally low in several statements. We found that nearly two-thirds (64.6%) of the participants had never heard of CTs. Similar to the present study, Al-Lawati et al. (Oman) and Al Rawashdeh et al. (KSA) found that their study participants had a low level of awareness about CTs [16,17]. Interestingly, a survey conducted in 2022 in the USA reported that a lower proportion (41.7%) of participants was unaware of CTs [22]. A recent survey conducted during the COVID-19 pandemic in the KSA by Alqahtani et al. also supports our findings. In their study, the awareness results concerning ethics committees and consent requirements were similar to the present study [23].

We categorized the knowledge score as per Bloom’s criteria [17,21]. The present study showed that most of the northern Saudi general population had either low (57.1%) or moderate (29.6%) overall knowledge scores. Some recently concluded surveys also reported similar findings [20,24,25]. However, a Jordanian study and a study conducted in the USA found that most of their study participants had moderate to good knowledge of CTs [22,26]. Possible variations between studies could be attributed to the differences including the study settings, sociocultural factors, and survey tools. We found that level of education was a significant factor associated with knowledge of CTs (*p* = 0.031). Interestingly, we did not find any other sociodemographic characteristics related to the participants’ knowledge. Similarly, Altaf et al. and Awwad et al. reported that people with higher education had significantly better knowledge of CT than others [20,26]. In contrast, Al Rawashdeh et al. and Ahram et al. reported that knowledge was significantly associated with other factors, namely, age group, gender, income, and employment status [17,27]. However, their report of an association with previous participation in health-related research was similar to our study.

A positive attitude is essential among the population to participate in CTs. It can encourage people to participate in CTs. Furthermore, a positive attitude is similar to infections and spreads among other family and community members [28,29]. However, we found that most of the participants had a low (73.5%) or moderate attitude towards CTs (14.8%). Our findings are supported by some studies in the KSA and other parts of the world, although a few studies reported a moderately positive attitude towards CTs [16,18,30,31]. This indicates that a poor attitude towards participating in CTs is a global phenomenon and needs immediate attention from stakeholders. Our population-based study revealed that a positive attitude was significantly higher among unmarried participants (*p* = 0.035) and the presence of chronic diseases (*p* = 0.008). Similar to the present study, several authors found that the presence of chronic diseases such as cancer might be a significant factor for participating in a CT [17,32]. This could be due to the increased sensitization among the patients, who might be looking for better treatment for their chronic diseases. Although CTs among healthy volunteers is equally important, as they can act as comparative groups and for prevention-based trials, the attitude among them is very low [33,34]. Another important factor revealed by the present study was marital status. A possible explanation is that currently married people might be thinking of commitments with family in the event of negative consequences due to the fact of their participation in a CT. The current survey found a positive correlation between participants’ knowledge and attitude scores (*p* < 0.001, rho = 0.329). The results of the present study in this context were similar to other studies, which also indicated a positive correlation between the population’s knowledge and attitude [17,26].

We performed this population-based cross-sectional survey in northern Saudi during the COVID-19 era with a standard and validated tool. However, the current survey has some limitations. First, we applied a questionnaire-based cross-sectional design, and the constraints related to this design must be considered while interpreting the findings of the current survey, such as recall bias and exaggerated responses. Next, we utilized a consecutive sampling method. Hence, the possibility of selection bias cannot be avoided. In addition, due to the wide sociocultural variation across the country (KSA), the present research findings cannot be generalized to the entire Saudi general population and other Middle East countries. Finally, the utilized study design attempted to find the association not the causation.

## 5. Conclusions

The present study revealed that most of the population has poor knowledge and moderate attitudes towards CTs. The knowledge categories were significantly associated with educational level, and the attitude categories were significantly associated with the presence of chronic diseases and marital status. Furthermore, we found a positive correlation between knowledge and attitude scores. Hence, we recommend improving the public’s knowledge of the importance of CT participation. This can be achieved through targeted health education programs at different public places. Such health education programs would improve the perceptions and attitude of the general population to participate in CTs, which is much needed for new drug development. Considering the present study’s limitations, we suggest an exploratory and mixed-methods survey in other regions of KSA to recognize region-specific health education needs.

## Figures and Tables

**Table 1:** Background and health-related characteristics of the participants (*n* = 480).

	Variable	Number (*n*)	Percentage (%)
Age group (years)	Less than 30 years 30 to 45 years Above 45 years	190 165 125	39.6 34.4 26.0
Gender	Male Female	305 175	63.5 36.5
Marital status	Married Unmarried *	361 119	75.2 24.8
Employment status	Public sector job Private job/self-employed/business Unemployed Retired	195 111 102 72	40.6 23.1 21.3 15.0
Education level	Up to high school University level	179 301	37.3 62.7
Income **	Less than SAR 5000 SAR 5000 to 10,000 More than SAR 10,000	161 172 147	33.5 35.8 30.6
Presence of any chronic diseases	Yes No	124 356	25.8 74.2
Previous participation in health research	Yes No	73 407	15.2 84.8

* Unmarried: single, divorced, separated, and widowed. ** USD 1 = 3.75.

**Table 2:** Responses of the participants for the knowledge items (*n* = 480).

	Item	Number (*n*)	Percentage (%)
Have you ever heard about clinical trials (CTs)?	Yes No/not sure	170 310	35.4 64.6
Definition of a CT	Correct answer Wrong answer	192 288	40.0 60.0
Have you heard of an ethics review committee for a CT?	Yes No	93 387	19.4 80.6
Have you heard about the Saudi FDA?	Yes No	270 210	56.3 43.7
Does the general authority of food and drug administration have any role in CT’s policy?	Yes No	376 104	78.3 21.7
Are there any ethical guidelines to regulate CTs?	Yes No	398 82	82.9 17.1
Is there a direct benefit to the participants in CTs?	Correct answer Wrong answer	98 382	20.4 79.6
Is there a direct benefit to the Saudi community due to the people participating in CTs?	Yes No	415 65	86.5 13.5
When can the research team start a CT?	Correct answer Wrong answer	235 245	49.0 51.0
Can the research team initiate the CT without the consent of the participants?	Yes No	436 44	90.8 9.2
Are the participants free to withdraw from the CT at any time?	Yes No	312 168	65.0 35.0
Patients’ confidential information (such as name) can be disclosed in the published article?	Yes No	183 297	38.1 61.9
	Total knowledge score (mean ± SD)	7.10 ± 2.03	

**Table 3:** Participants’ responses in each attitude section (*n* = 480).

	Item	Number (*n*)	Percentage %
Do you agree to test a new experimental non-approved drug on patients?	Yes No/not sure	116 364	24.2 75.8
Do you agree to test an already approved drugs on patients?	Yes No/not sure	320 160	66.7 33.3
Do you agree to test a new experimental drug on healthy volunteers?	Yes No/not sure	256 224	53.3 46.7
Do you agree to test a new experimental drug on child patients?	Yes No/not sure	57 423	11.9 88.1
Do you agree to test an already approved drug on child patients?	Yes No/not sure	221 259	46.0 54.0
Are you willing to participate in a CT?	Yes No/not sure	56 424	11.7 88.3
Do you want to know more about CT?	Yes No	359 121	74.8 25.2
What is your perception towards CT?	Positive answer Negative answer	309 171	64.4 35.6
Would you trust the research team which is conducting the CT?	Yes No/not sure	360 120	75.0 25.0
	Total attitude score	4.38 ± 2.04	

**Table 4:** Participants’ characteristics and their association with knowledge and attitude categories (*n* = 480). Test applied: chi-square test.

Variable			Knowledge			Attitude		
		Total	Low and Moderate (*n* = 416)	Excellent (*n* = 64)	*p*-Value	Low and Moderate (*n* = 424)	Excellent (*n* = 56)	*p*-Value
Age group (years)	Less than 30 years 30 to 45 years Above 45 years	190 165 125	159 (83.7) 149 (90.3) 108 (86.4)	31 (16.3) 16 (9.7) 17 (13.6)	0.187	163 (85.8) 149 (90.3) 112 (89.6)	27 (14.2) 16 (9.7) 13 (10.4)	0.366
Gender	Male Female	305 175	266 (87.2) 150 (85.7)	39 (12.8) 25 (14.3)	0.369	268 (87.9) 156 (89.1)	37 (12.1) 19 (10.9)	0.397
Marital status	Married Unmarried	361 119	314 (87.0) 102 (85.7)	47 (13.0) 17 (14.3)	0.415	326 (90.3) 98 (82.4)	35 (9.7) 21 (17.6)	0.019 *
Employment status	Public sector job Private job/self-employed Unemployed Retired	195 111 102 72	173 (88.7) 92 (82.9) 91 (89.2) 60 (83.3)	22 (11.3) 19 (17.1) 11 (10.8) 12 (16.7)	0.512	175 (89.7) 100 (90.1) 85 (83.3) 64 (88.9)	20 (10.3) 11 (9.9) 17 (16.7) 8 (11.1)	0.461
Education level	Up to high school University or higher	179 301	165 (92.2) 251 (83.4)	14 (7.8) 50 (16.6)	0.006 *	158 (88.3) 266 (88.4)	21 (11.7) 35 (11.6)	0.973
Income ** (monthly)	Less than SAR 5000 SAR 5000 to 10,000 More than SAR 10,000	161 172 147	137 (85.1) 145 (84.3) 134 (91.2)	24 (14.9) 27 (15.7) 13 (8.8)	0.154	143 (88.8) 152 (88.4) 129 (87.8)	18 (11.1) 20 (11.6) 18 (12.2)	0.958
Presence of any chronic diseases	Yes No	124 356	103 (83.1) 313 (87.9)	21 (16.9) 43 (12.1)	0.113	101 (81.5) 323 (90.7)	23 (18.5) 33 (9.3)	0.006 *
Previous participation in health research	Yes No	73 407	54 (74.0) 362 (88.9)	19 (26.0) 45 (11.1)	0.005 *	60 (82.2) 364 (89.4)	13 (17.8) 43 (10.6)	0.206

* Significant value (*p* < 0.05). ** 1 USD = SAR 3.75.

**Table 5:** Factors associated with the northern Saudi general population’s knowledge and attitude towards CTs. The test applied: binomial logistic regression analysis (*n* = 480).

Variables		Knowledge (Poor and Moderate vs. Excellent)		Attitude (Poor and Moderate vs. Excellent)	
		Adjusted Odds Ratio (AOR) **/95% Confidence Interval (CI)	*p*-Value	AOR ** (95% CI)	*p*-Value
Age group (years)	Less than 30 years 30 to 45 years Above 45 years	Ref. 0.56 (0.29–1.09) 0.79 (0.41–1.54)	0.088 0.500	Ref. 0.66 (0.38–1.31) 0.73 (0.35–1.50)	0.238 0.389
Gender	Male Female	Ref. 1.12 (0.63–2.01)	0.704	Ref. 1.02 (0.54–1.91)	0.960
Marital status	Married Unmarried	Ref. 1.14 (0.61–2.11)	0.689	Ref. 1.84 (1.17–2.98)	0.035 *
Employment status	Public sector job Private job/self-employed Unemployed Retired	Ref. 1.24 (0.50–3.06) 0.98 (0.44–2.18) 1.48 (0.58–3.74)	0.637 0.951 0.411	Ref. 0.48 (0.19–1.20) 0.54 (0.26–1.15) 0.55 (0.21–1.44)	0.117 0.111 0.225
Education level	Up to high school University or higher	Ref. 1.73 (1.15–2.84)	0.031 *	Ref. 1.15 (0.60–2.18)	0.677
Income (monthly)	Less than SAR 5000 SAR 5000 to 10,000 More than SAR 10,000	Ref. 0.62 (0.31–1.19) 1.58 (0.79–2.74)	0.148 0.728	Ref. 1.06 (0.57–1.98) 0.91 (0.53–2.11)	0.854 0.512
Presence of any chronic diseases	Yes No	Ref. 0.66 (0.37–1.18)	0.159	Ref. 0.71 (0.54–0.91)	0.008 *
Previous participation in health research	Yes No	Ref. 0.63 (0.47–0.89)	0.007 *	Ref. 0.49 (0.23–1.04)	0.062

* Significant value (two-tailed). ** Adjusted variables in logistic regression (enter method) age group, gender, married status, employment status, education level, income, presence of chronic diseases, and previous participation in health research.

**Table 6:** Correlation between participants knowledge and attitude towards CT (*n* = 480). Test applied: Spearman’s correlation test.

	Spearman’s Coefficient Value (rho)	*p*-Value
Knowledge–Attitude	0.329	<0.001 *

* Significance: *p*-value < 0.05 (two-tailed test).

## Data Availability

The data used in this study will be provided by the principal investigator upon request.
